# Proximal Myopathy due to m.5835G>A Mutation in Mitochondrial MT-TY Gene

**DOI:** 10.1155/2018/8406712

**Published:** 2018-12-10

**Authors:** C. Simoncini, V. Montano, G. Alì, R. Costa, G. Siciliano, M. Mancuso

**Affiliations:** ^1^Department of Clinical and Experimental Medicine, Neurological Clinic, University of Pisa, Pisa, Italy; ^2^Division of Pathological Anatomy, University of Pisa, Pisa, Italy; ^3^Department of Biomedical and Neuromotor Sciences (DIBINEM), University of Bologna, Bologna, Italy

## Abstract

Mitochondrial (mt) tRNA (MTT) gene mutations are an important cause of mitochondrial diseases and are associated with a wide range of clinical presentations. Most mutations fall into three mitochondrial tRNAs (tRNAIle, tRNALeu (UUR), and tRNALys) and are responsible for half of the mitochondrial diseasees associated with tRNA mutation, with MERRF, MELAS, mitochondrial myopathy, and Leigh syndrome being the most frequent phenotypes. More than 100 tRNA pathogenetic mutations are described, showing little correlation between the observed clinical phenotype and a specific mitochondrial tRNA mutation. Furthermore different mutation can manifest with similar clinical phenotypes, making the genotype-phenotype correlation difficult. Here we report the case of an Italian 53-year-old woman presenting with a proximal myopathy and the m.5835G>A mutation in MT-TY gene coding for the mitochondrial tRNA Tyrosine gene.

## 1. Introduction

Mitochondrial (mt) tRNA (MTT) gene mutations are an important cause of mitochondrial diseases and are associated with a wide range of clinical presentations, ranging from isolated myopathy to multisystem disorders with encephalopathy, diabetes, hearing loss, dysphagia, and cardiomyopathy [1]. Today how MTT mutations cause mitochondrial disease is unknown, and it is not clear why patients with the same mutation exhibit different phenotypes, whereas other patients manifest clinically identical syndromes [2]. Several mutations in the MT-TY gene coding for tRNA Tyrosine have been reported (https://www.mitomap.org), with clinical phenotypes ranging from chronic external ophthalmoplegia to cardiomyopathy (Table 1). Here we describe a case of mitochondrial myopathy with the m.5835G>A mutation in MT-TY gene coding for tRNA Tyrosine. A revision of the clinical presentations caused by MT-TY different gene mutations is also presented.

## 2. Case Report

**Figure 1:**
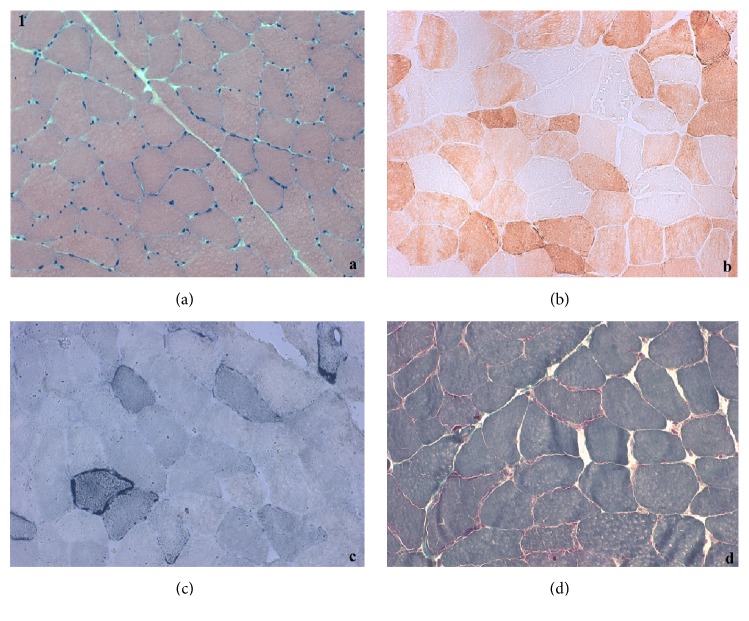
Muscle biopsy: (a) hematoxylin eosin; (b) staining for cytochrome oxidase; (c) Succinate dehydrogenase (SDH) staining; (d) Gömöri trichrome stain.

**Figure 2:**
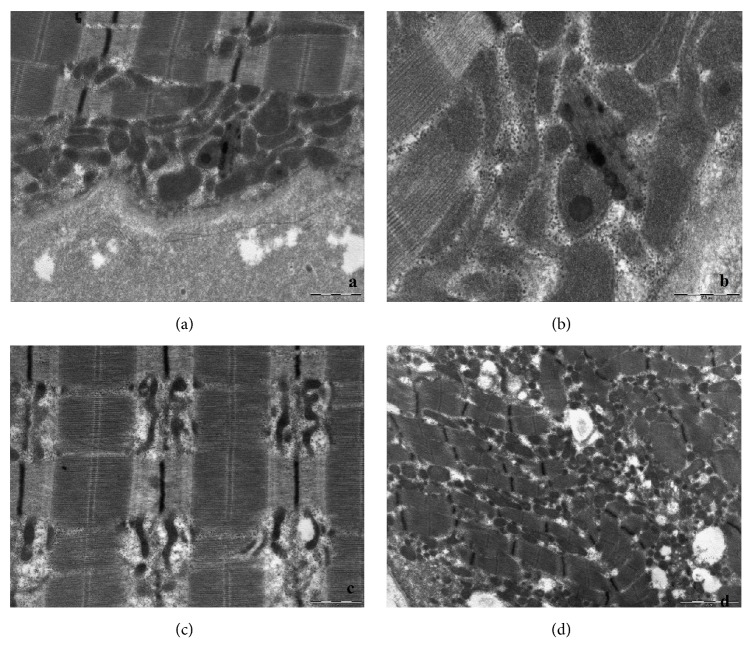
Ultrastructural examination on muscle: (a) and (b) show intra mitochondrial lipid inclusion; (c) mitochondria with transversal orientation to the myofiber; (d) mitochondrial hyperplasia.

A 53-year-old Italian woman came to our attention for a two-year history of progressive lower limbs weakness with difficulty in climbing stairs and posture changes; the patient also complained of occasional difficulty in swallowing. She presented mild hyperCkemia (300 U/L), with isolated occurrence of higher values (up to 1800 U/L) after exercise. Patient's parents were not consanguineous. She had family history of diabetes and cardiac conduction disorders (mother, who died at age 85) and ischaemic heart disease (father, who died at age 90). Her personal history was unremarkable. Neurological examination showed mild proximal weakness of lower and upper limbs and mild neck flexor muscles weakness (MRC 4/5). Forearm ischaemic test revealed basal hyperlactacidemia (42 mg/dL, reference value 4,5-19,8 mg/dl). Electromyography showed a myopathic pattern. Muscular CT showed mild left femoral quadriceps and paravertebral muscles hypotrophy. Cardiological evaluation and spirometry were both normal. Muscle biopsy revealed myopathic changes with scattered ragged red and blue fibers, as well as COX negative fibers (Figure 1). Ultrastructural examination on muscle confirmed the mitochondrial alterations with mitochondrial hyperplasia and ring cristae, intramitochondrial lipid inclusion, and mitochondria with transversal orientation to the myofiber (Figure 2).

Muscular mtDNA sequencing showed the heteroplasmic mutations m.5835G>A in MT-TY gene, coding for tRNA Tyrosine. The mutation was heteroplasmic in skeletal muscle; unfortunately, we could not test for the mutation in other peripheral tissues.

## 3. Discussion

Mitochondrial DNA (mtDNA) encodes for 22 tRNA genes, one for each of 20 amino acids, and two for tRNA Leu (UUR and CUN codons) and tRNA Ser (UCN and AGY codons); the products of these tRNA are necessary for the correct functioning of mitochondria [3]. More than half of the reported mtDNA mutations are located in a tRNA gene; thus, mitochondrial tRNA genes are hot spots for mitochondrial disease [2, 4, 5]. Di Mauro and Schon in 2001 defined the pathogenic criteria for mtDNA point mutations, which are absence in healthy control, alteration of an evolutionary conserved residue, heteroplasmy, segregation with disease and correlation of heteroplasmy degree with clinical severity and cell pathology as documented by single-fiber polymerase chain reaction (PCR), and demonstration of defects of mitochondrial protein synthesis and respiratory chain deficiencies affected tissues [6].

Our patient, with a phenotype of proximal myopathy, harboured the m.5835G>A mutation in MT-TY gene. This mutation was already described in 2008 by Kornblum, who reported a 22-year-old male patient of Turkish ethnic origin with chronic progressive external ophthalmoplegia and exercise intolerance [7]. The mutation causes a C→U transition at an evolutionary highly conserved position in the T-stem of the tRNATyr. Kornblum proved its pathogenicity by muscle single fiber PCR analysis that showed a high (98%) amount of m.5835G>A mutation in all SDH-positive COX negative fibers demonstrating a high threshold for phenotypical expression on the single cell level. Besides, steady state tRNA levels performed with northern blot in the patient's skeletal muscle revealed 95% reduction of the tRNATyr amount in total skeletal muscle RNA [7].

We have considered this mutation to be pathogenetic because both MITOMAP and Yahram scoring support its pathogenicity. In particular, MITOMAP counts the mutation between the mtDNA coding region and RNA sequence variants; the variant has a pathogenicity scoring from MitoTIP of 12,9715 (54,7%), i.e., “possibly pathogenetic” [8]. Moreover, the mutation causes a C→U transition at an evolutionary highly conserved position in the T-stem of the tRNATyr. Considering our data and the studies on biochemical evaluation of respiratory chain activities, single fiber analysis and steady state of the G5835A MT-TY variant performed by Kornblum et al. [7], the mutation has a pathogenicity score according to the scoring system of Yarham et al. (2011) of 18/20, i.e., “definitely pathogenetic” [9]. Therefore, since both the MITOTIP and the Yarham scores are high, the mutation should be considered pathogenetic.

Many other mutations in mitochondrial MTTY gene have been reported in literature, predominantly exhibiting a myopathic phenotype (summarized in Table 1). Pulkes et al. in 2000 described a patient harboring a novel heteroplasmic m.5874A-G somatic mutation presenting with exercise intolerance, mild bilateral ptosis, limb weakness, and respiratory chain complex III deficiency, hypnotizing that the phenotype associated with this mutation is similar to cytochrome b deficiency because of a high content of tyrosine in cytochrome b and COX III subunits [10]. Sahashi and Raffelsberger in two different papers in 2001 described two patients presenting with chronic progressive external ophthalmoplegia, mild myopathy, and exercise intolerance and, respectively, a single base pair deletion at position 5885 (5885delT) and a m.5877G>A transition; both mutations were somatic and affected the tRNA functions altering its secondary and tertiary structure [11, 12].

Scaglia in 2003 described a child harboring the homoplasmic m.5843A>G transition in the MTTY gene with a history of steroid-resistant nephrotic syndrome caused by focal segmental glomerulosclerosis at age of 2 years and onset of dilated cardiomyopathy from the age of 7 years; combined respiratory chain defect and a partial coenzyme Q10 deficiency were found on skeletal muscle biopsy. Also the patient's mother, symptomatic for migraine headaches, was homoplasmic for the m.5843A>G mutation, suggesting that other factors, maybe epigenetic, could explain the phenotypic variability of mutation between the proband and her mother [13].

This second patient with m.5835G>A, clinically presenting proximal limb girdle myopathy, confirms the pathogenicity of the mutation, expanding the clinical manifestation of this nucleotide change.

## Figures and Tables

**Table 1:** Overview of the published cases with mitochondrial MTTY gene mutations.

Nucleotide Change	Phenotype	Reference
m.5874A>G	Ptosis, limb weakness, exercise intolerance with complex III deficiency	Pulkes T, Neurology 2000
m.5885delT	Chronic progressive external ophthalmoplegia, myopathy and exercise intolerance	Raffelsberger T, Neurology 2001
m.5877G>A	Chronic progressive external ophthalmoplegia, proximal muscle weakness	Sahashi T, Journal of medical genetics 2001
m.5843A>G	Focal segmental glomerulosclerosis, cardiomyopathy	Scaglia F, American journal of medical genetics 2003
m.5835G>A	Chronic progressive external ophthalmoplegia	Kornblum C Bioscience reports 2008
m.5835G>A	Limb girdle muscle weakness, swallowing impairment	Present case
